# Right Temporoparietal Junction Plays a Role in the Modulation of Emotional Mimicry by Group Membership

**DOI:** 10.3389/fnhum.2021.606292

**Published:** 2021-02-10

**Authors:** Shenli Peng, Beibei Kuang, Ling Zhang, Ping Hu

**Affiliations:** ^1^College of Education, Hunan Agricultural University, Changsha, China; ^2^School of International Relations, National University of Defense Technology, Nanjing, China; ^3^Department of Psychology, Renmin University of China, Beijing, China

**Keywords:** rTPJ, emotional mimicry, group membership, eletromyography, emotion

## Abstract

Our prior research demonstrated that the right temporoparietal junction (rTPJ) exerted a modulatory role in ingroup bias in emotional mimicry. In this study, two experiments were conducted to further explore whether the rTPJ is a neural region for emotional mimicry or for the modulation of emotional mimicry by group membership in a sham-controlled, double-blinded, between-subject design. Both experiments employed non-invasive transcranial direct current stimulation (tDCS) to temporarily change the cortical excitability over the rTPJ and facial electromyography (fEMG) to measure facial muscle activations as an index of emotional mimicry. After the anodal or sham stimulation, participants in Experiment 1 passively viewed a series of happy clips, while participants in Experiment 2 viewed happy clips performed by ethnic ingroup and outgroup models. fEMG analyses revealed that participants in Experiment 1 showed the same degree of happy mimicry for both tDCS conditions (anodal vs. sham) and participants in Experiment 2 showed an ingroup bias in happy mimicry in the sham condition, which disappeared in the anodal condition. Taken together, the present study demonstrated that rTPJ plays a role in the modulation of emotional mimicry by group membership.

Many researches have documented that people are more likely to unconsciously mimic an expression when it is shown on an ingroup member's face than when it is shown on an outgroup member's face (Bourgeois and Hess, 2008; van der Schalk et al., 2011; Hühnel, 2015; Kuszynski, 2015). This phenomenon is characterized as ingroup bias in emotional mimicry, supporting the contextual model (Hess and Fischer, 2014).

To date, however, why people show this ingroup bias remains an issue. Our recent study (Peng et al., 2020) took the first step by showing that the right temporoparietal junction (rTPJ) could modulate ingroup bias in emotional mimicry. Specifically, we recruited a group of college students and randomly assigned them to three transcranial direct current stimulation (tDCS) conditions: anodal, cathodal, and sham. After temporary stimulation over the rTPJ, participants passively viewed a series of video clips depicting different emotions (happiness and anger) that were performed either by ethnic ingroup or outgroup models. Using the facial electromyography (fEMG), facial muscle activations of the *zygomaticus major* (ZM) and *corrugator supercilii* (CS) were recorded simultaneously to index the mimicry of happiness and anger, respectively. In the sham condition, an ingroup bias in mimicry of happiness was found, which was in accordance with previous studies. Interestingly, we further found that this bias disappeared in both anodal and cathodal conditions, indicating the modulatory role of the rTPJ. We attributed our results to the possibility that self–ingroup (vs. self–outgroup) overlap in mental representations changed accompanied with a change in cortical excitability over the rTPJ during tDCS implementation. This was in line with the idea that self–other overlap is responsible for ingroup bias in emotional mimicry (Hühnel et al., 2018) and other areas such as empathy (Ellemers and Haslam, 2012). However, our data could not rule out the possibility that the rTPJ served as a neural substrate of emotional mimicry. That is, activation of rTPJ would result in increased emotional mimicry, irrespective of group membership.

Generally, there are two possibilities in terms of the role of the rTPJ in the modulatory control of automatic facial responses. The first is that rTPJ acts as a core area associated with mimicry of different emotional expressions. Gamond and Cattaneo (2016) employed transcranial magnetic stimulation (TMS) to explore the neural underpinnings of the ingroup bias in emotion recognition and found that the dorsomedial prefrontal cortex (dmPFC) was responsible for this ingroup bias, while the rTPJ mainly affected emotion discrimination, instead of group membership differentiation. Consistently, Rauchbauer et al. (2016) revealed distinct neural areas engaged in the modulation of behavioral mimicry by group membership and emotion. Participants had to perform a social-affective mimicry task with their brain activations simultaneously recorded by functional magnetic resonance imaging (fMRI). In this task, participants were instructed to lift their right index or middle finger according to the number cue presented on an image of a hand that mirrored their own hand. Simultaneous to the presentation of the number cue, the hand stimulus showed a finger lifting movement, which displayed either congruent or incongruent finger movement to the cued finger. Additionally, either an ingroup or outgroup female face, who displays either a happy or angry emotion, was presented above the hand and number cue. Automatic imitation behavior was reflected by the mean reaction time difference between incongruent and congruent trials. Results revealed that the rTPJ was one of the areas responsible for the modulation of mimicry by emotional expression, and mimicry modulation by group membership was associated with the left ventral premotor cortex, inferior parietal lobule, bilateral anterior insula, and mid-cingulate cortex. These two studies suggest that the rTPJ is associated with the modulation of facial mimicry by emotional expressions.

However, there are inconsistent findings in research on neural regions of emotional mimicry (Lee et al., 2006; Likowski et al., 2012; Rymarczyk et al., 2018; Korb et al., 2019). Lee et al. (2006) demonstrated that imitation of emotional facial expressions was related to increased activation in the bilateral inferior frontal gyrus rather than the rTPJ. In a recent study, Rymarczyk et al. (2018) recorded the neural correlates underlying automatic emotional mimicry and revealed distinct neural regions associated with motor simulation of facial expression and emotional processing. The former regions included major areas (i.e., the inferior frontal gyrus) of the classic mirror neuron system (MNS), while the latter regions contained the insula and amygdala as part of the extended MNS. None of these studies have exhibited the rTPJ as a candidate substrate for the mimicry of emotional expressions. Thus, it remains an open question whether the rTPJ can bring forth modulatory control of emotional mimicry.

Another possibility is that the rTPJ is responsible for differentiating between ingroup and outgroup members, that is, the rTPJ is associated with the modulation of emotional mimicry by group membership. This idea is in accordance with prior theoretical and empirical studies demonstrating that the rTPJ is accountable for intergroup differences in perception (Harris and Fiske, 2006), judgment (Freeman et al., 2010), and parochial altruism (Baumgartner et al., 2012). For example, there is a positive relationship between the volume of the rTPJ and increased impartiality in intergroup conflict (Baumgartner et al., 2012), favoring the modulatory role of the rTPJ in intergroup bias in social cognition. Additionally, Wang and Hamilton (2012) proposed a social top-down response modulation (STORM) model to illustrate information processing during mimicry. The STORM model claimed that mimicry is guided and monitored by social information, which involves the interaction between the mirror system and mentalizing system. As the rTPJ is the core area of the mentalizing system, this model implied the role of the rTPJ in processing social information during mimicry. In line with this idea, left TPJ was found increasingly activated when facial mimicry occurred for ingroup members (de Klerk et al., 2019).

## Overview of the Present Study

As reviewed earlier, although our prior study (Peng et al., 2020) revealed alteration in rTPJ activity resulted in change of facial mimicry toward ethnic ingroup and outgroup expressions, it left an open question what the precise role rTPJ exerted in this modulation. The present study aimed to tap this issue by testing the two possible explanations via two experiments. Experiment 1 explores whether mimicry of emotional faces (e.g., happiness) is modulated by the rTPJ. Experiment 2 examines whether mimicry of ingroup vs. outgroup emotional faces is regulated by the rTPJ. Both experiments employed non-invasive tDCS to temporarily alter the cortical excitability over the rTPJ and investigate its effect on the subsequent facial emotional mimicry performance in a sham-controlled, double-blinded, between-subject design. A discrepancy between the two experiments is that participants in Experiment 1 were shown only happy faces of their own-race models, while participants in Experiment 2 were presented with happy faces from ethnic ingroup and outgroup models. Based on the literature review, the authors hypothesized that the rTPJ is associated with the modulation of facial emotional mimicry by group membership. Therefore, Experiment 1 hypothesizes that excitation of the rTPJ has no effect on the mimicry of happy faces without information about group membership, that is, fEMG activations in the anodal and sham conditions are equivalent. Experiment 2 hypothesizes that temporary alteration of the rTPJ activity modulates ingroup bias in happy faces mimicry, that is, an ingroup bias of happy mimicry will show in the sham condition and disappear in the anodal condition.

### Participants

There were 42 and 44 randomly recruited college students from a university in Mainland China who participated in Experiment 1 and Experiment 2, respectively. All participants had normal or corrected-to-normal eyesight, and none of them reported a history of neurological or psychiatric disorders. Participants in both experiments were randomly assigned to two groups (see details in Table 1). Both experiments were approved by the local ethics committee, and written consent was obtained from each participant.

**Table 1:** Demographic statistics for Experiments 1 and 2.

	**Experiment 1**		**Experiment 2**	
	***N***	**Age (*M* ± SD)**	***N***	**Age (*M* ± SD)**
Anodal	21 (7 males)	18.9 ± 0.8	22 (7 males)	22.0 ± 1.3
Sham	21 (7 males)	19.3 ± 1.0	22 (7 males)	21.4 ± 1.6

### Materials

The materials of the present study were dynamic emotional clips derived from the 3D dynamic facial expression database developed by Yin et al. (2008). Experiment 1 selected a total of 20 happy clips performed by 20 East Asian models (8 females), and Experiment 2 selected a total of 40 happy clips performed by 20 East Asian models (8 females) and 20 Western Caucasian models (8 females). All clips were edited to the same 1,000-ms duration, changing from a neutral expression at the beginning to the full-blown emotional expression by the end. The stimuli were validated in our prior work (Peng et al., 2020).

### tDCS Protocol

tDCS was performed using a DC-STIMULATOR PLUS (neuroCare Group, Germany). It was delivered through a pair of 35 cm^2^ sponge electrodes soaked in saline. The site for the rTPJ was the midpoint of CP6 and P6, according to the International 10–20 electroencephalography (EEG) system, with the reference electrode placed over the left cheek. In the anodal condition, a weak current (1.5 mA) was delivered for 20 min. In the sham condition, the current lasted for only 30 s, although the electrode was in place for 20 min. This procedure, according to prior research (Nitsche et al., 2003), made the participants “feel the initial itching sensation on the scalp at the beginning but received no current for the rest of the stimulation period” (Bardi et al., 2017), thus allowing us to blind participants for the respective stimulation types. The fade-in and fade-out times for each condition were both 15 s. It should be noted that both participants and research assistant, who had been trained for the use of the apparatus, were blinded to the research purpose and stimulus condition. After the stimulation, the tDCS electrodes were removed, and participants were taken to the EMG laboratory to wear EMG electrodes by the researcher (the first author). The time between the end of tDCS stimulation and emotional mimicry task was about 10 min.

### Procedure

Both experiments adopted a passive viewing paradigm to induce emotional mimicry. After the tDCS administration, participants were briefly introduced to the experimental procedure. A cover story stated that this study was designed to rate the emotions performed by various models to build a dynamic, happy face database. Here, ethnicity was made salient, as in prior studies (van der Schalk et al., 2011; Peng et al., 2020), for participants in Experiment 2. In the formal procedure, each trial began with a fixation (300–500 ms) and then with a happy clip (1,000 ms). Participants were instructed to view the clip carefully and rate its valence on the next display (data not shown). The order of all the clips was randomized for each participant. The intertrial interval was 1,000–1,200 ms.

### Data Acquisition and Analysis

Biopac system EMG (BIOPAC Systems, Inc., Santa Barbara, CA, USA) was employed to record the fEMG activities. Placement of the ZM, CS, and reference electrode was in accordance with prior research (Fridlund and Cacioppo, 1986; Deng and Hu, 2018). The fEMG activation was recorded at 2,048 Hz with a 28–500 Hz band-pass filter.

Raw data were transferred into fEMG signals by calculating the root mean squares (RMS) on AcqKnowledge 5.0. The fEMG activations were expressed as change in activity in microvolts from the pre-stimulus level, defined as the mean activity during the last second before the stimulus onset. The fEMG (ZM and CS) activations were averaged under various conditions. In Experiment 1, a tDCS (anodal vs. sham) × muscle (ZM vs. CS) mixed ANOVA was conducted on fEMG responses. In Experiment 2, a tDCS (anodal vs. sham) × group (ingroup vs. outgroup) × muscle (ZM vs. CS) mixed ANOVA was conducted. One-sample two-tailed *t*-tests against zero were conducted for various conditions to determine whether fEMG responses differed from baseline.

## Results

### Experiment 1

**Figure 1:**
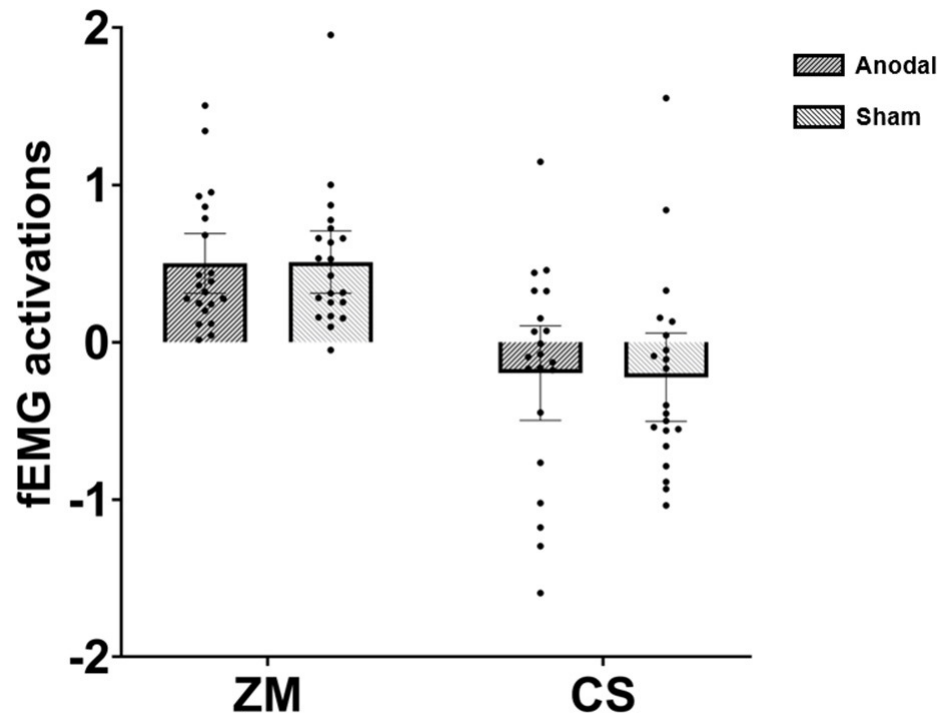
Facial electromyography (fEMG) [*zygomaticus major* (ZM) and *corrugator supercilii* (CS)] activations under various conditions in Experiment 1; dots refer to individual datapoints.

The mixed ANOVA revealed a significant main effect of muscle, *F* (1, 40) = 31.49, *p* < 0.001, η^2^ = 0.44. As depicted in Figure 1, further analysis showed that happy faces induced greater ZM activation (*M* = 0.51) relative to CS activation (*M* = −0.21), 95%CI = (0.46, 0.97). One-sample two-tailed *t*-tests against zero indicated that ZM responses to happiness were significantly larger than zero in both anodal [*t* (20) = 5.51, *p* < 0.001, 95%CI = (0.31, 0.69)] and sham [*t* (20) = 5.39, *p* < 0.001, 95%CI = (0.31, 0.71)] conditions. In summary, Experiment 1 demonstrated that participants in the anodal and sham groups displayed an equivalent degree of happy mimicry.

### Experiment 2

The mixed ANOVA revealed significant main effects of muscle [*F* (1, 42) = 234.83, *p* < 0.001, η^2^ = 0.85] and tDCS [*F* (1, 42) = 4.26, *p* = 0.045, η^2^ = 0.09], which were qualified by the significant effects of tDCS × group [*F* (1, 42) = 6.30, *p* = 0.016, η^2^ = 0.13] and group × muscle [*F* (1, 42) = 5.83, *p* = 0.02, η^2^ = 0.12). For the former interactive effect, further analysis suggested that ingroup (relative to outgroup) happy faces induced greater fEMG activations in the sham condition [*p* = 0.003, 95%CI = (0.06, 0.28)], which was not the case in the anodal condition (*p* = 0.66). For the latter interactive effect, further analysis indicated that ZM response for ingroup happy faces was larger than for outgroup happy faces, while CS responses were not different between ingroup and outgroup faces. One-sample two-tailed *t*-tests against zero demonstrated that ZM response to ingroup in the sham condition was larger than zero, *t* (21) = 7.11, *p* < 0.001, and ZM responses to both ingroup [*t* (21) = 5.85, *p* < 0.001] and outgroup happiness [*t* (21) = 7.22, *p* < 0.001] in the anodal condition were also larger than zero. CS activations in all conditions were significantly smaller than zero (*p*_s_ < 0.001).

**Figure 2:**
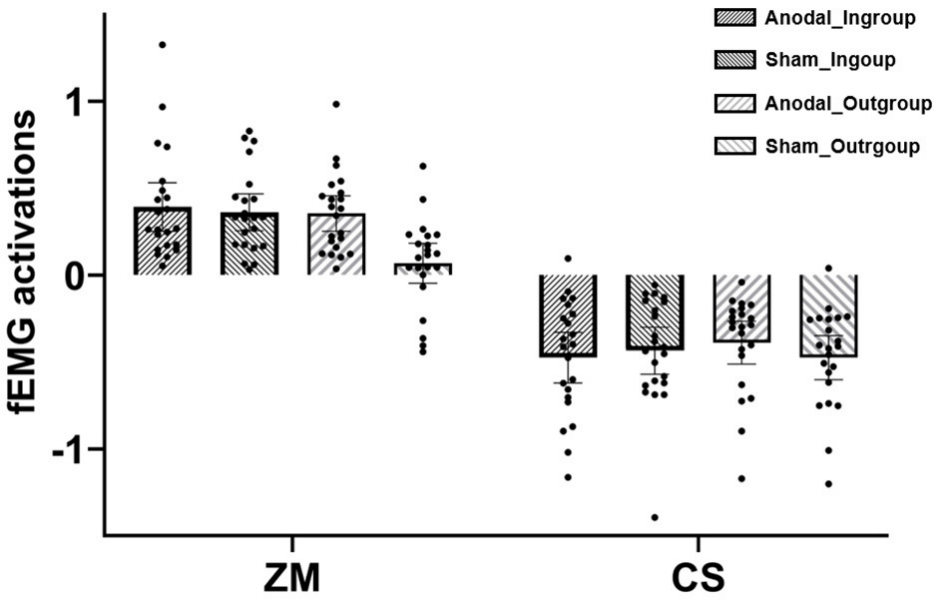
Facial electromyography (fEMG) [*zygomaticus major* (ZM) and *corrugator supercilii* (CS)] activations under various conditions in Experiment 2; dots refer to individual datapoints.

Given the specialized index of ZM for happy mimicry (e.g., van der Schalk et al., 2011; Deng and Hu, 2018; Rymarczyk et al., 2018), a tDCS (anodal vs. sham) × group (ingroup vs. outgroup) mixed ANOVA was conducted on ZM activation, and the results uncovered significant main effects of group [*F* (1, 42) = 14.35, *p* < 0.001, η^2^ = 0.26] and tDCS [*F* (1, 42) = 5.77, *p* = 0.02, η^2^ = 0.12], which were qualified by the significant two-way interaction, *F* (1, 42) = 8.55, *p* = 0.006, η^2^ = 0.12. As shown in Figure 2, further analysis revealed that ZM response to ingroup (*M* = 0.36) was larger than to outgroup happiness (*M* = 0.07) in the sham condition, *F* (1, 42) = 22.53, *p* < 0.001, η^2^ = 0.35, while they did not differ in the anodal condition. This suggested that there was an ingroup bias in mimicry of happy faces in the sham condition, which was absent in the anodal condition.

## Discussion

The present study aimed to explore the exact role of the rTPJ in the modulation of ingroup bias in emotional mimicry, in the aid of non-invasive tDCS technique. Experiment 1 found the mimicry of happy faces (without explicit race-related information) in the sham condition was not different from that in the anodal condition, indicating rTPJ did not regulate mimicry activity of happy faces. When happy expression was presented on both ethnic ingroup and outgroup faces, Experiment 2 confirmed an ingroup bias in happy mimicry in the sham tDCS condition and further found this ingroup bias disappeared in the anodal tDCS condition, suggesting rTPJ modulated the ingroup bias in facial mimicry.

Experiment 1 reveals that enhanced cortical excitability over the rTPJ does not improve emotional mimicry. This finding aids the interpretation of our prior research (Peng et al., 2020) by ruling out the possibility that the rTPJ regulates facial mimicry toward different emotions. Many previous studies attempting to explore the neural bases for emotional mimicry have demonstrated that both the classical MNS and extended MNS are engaged in emotional mimicry, with the former linked to motor stimulation and the latter linked to the process of affective expressions (Likowski et al., 2012; Rymarczyk et al., 2018, 2019). While the core areas of the classical MNS constitute the inferior parietal lobule and inferior frontal gyrus, the extended MNS mainly includes the superior temporal sulcus, middle temporal gyrus, insula, amygdala, and somatosensory cortex. Neither of them involves the rTPJ, implying that it may not be the region responsible for automatic mimicry of emotional expressions. Thus, it is rational that altering cortical excitability does not change the level of happy mimicry, as shown in Experiment 1.

Experiment 2 suggests that mimicry of ingroup (vs. outgroup) happy faces is modulated by the rTPJ, revealing the exact role of the rTPJ in the modulation of ingroup bias in emotional mimicry. In addition to the classical and extended MNS associated with facial mimicry, there are other neural regions that may bring forth modulatory control over mimicry behavior (see Kraaijenvanger et al., 2017 for a review), lending support for the contextual view of emotional mimicry (Hess and Fischer, 2014). For example, selective facial mimicry of faces with direct gaze over faces with averred gaze is linked to activation over the posterior superior temporal sulcus (de Klerk et al., 2018). Consistent with our study, increased activation of the rTPJ associated with ingroup bias in facial mimicry is depicted in prior research (de Klerk et al., 2019). Given that much behavioral evidence indicates, although it occurs automatically, unconsciously, and unintentionally, emotional mimicry is heavily influenced by a series of top-down modulatory factors (Fischer and Hess, 2017), the current study provides neural evidence for this top-down process.

The role of the rTPJ in regulating aspects of social cognition has been extensively elucidated. For example, Baumgartner et al. (2012, 2014, 2015) showed that the rTPJ plays a causal role in the modulation of parochialism, a preference for altruistic behavior toward own-group members and a tendency for indifference, mistrust, or even hostility toward other-group members (Brewer, 1979). By means of TMS, Baumgartner et al. (2014) disrupted the function of the rTPJ and found diminished parochialism, which was achieved by blending outgroup into ingroup and reducing punishment of the outgroup. Additionally, researchers revealed a positive association between the volume of the rTPJ and reduced intergroup bias, providing a neuroanatomical basis for the role of the rTPJ in modulating intergroup bias (Baumgartner et al., 2012). Complementing these findings, the present study indicates that the rTPJ plays a modulatory role in intergroup bias in emotional mimicry.

Of particular interest, we would like to link the current study to prior research regarding the rTPJ's role in embodiment, a typical social cognition that is closely related to emotional mimicry (Arnold and Winkielman, 2020). Substantial brain stimulation studies have confirmed the link between the rTPJ and embodiment (Wang et al., 2016; Martin et al., 2019a, 2020). Especially, Martin et al. (2019b) revealed participants from Southeast Asian Singapore and Australia showed comparable embodiment mental rotation performance, and subsequently, these two culture groups showed equivalent embodied rotation performance after receiving anodal stimulation over the rTPJ. This research is enlightening for its implication on the culture difference vs. resemblance in embodiment and other social cognition. However, Martin et al. (2019b) did not enroll ethnic group membership in their study, for example, categorizing the avatar in embodied rotation task as ethnic ingroup and outgroup to examine the baseline performance and the subsequent tDCS (over the rTPJ) stimulation effect between two cultural groups. This investigation, the authors believed, should shed light into the cultural ingroup vs. outgroup differences in embodiment mental rotation as well as deeper understanding of the relationship between embodiment and emotional mimicry.

With regard to the tDCS stimulation, offline (preceding mimicry paradigm) rather than online (concurrent with mimicry paradigm) was selected in the current study for two reasons. First, the current study was a replication and further investigation based on our prior research (Peng et al., 2020), and thus, a design consistent with prior research was more appropriate. Second, previous work indicated that effects of (at least) anodal stimulation were more robust with offline stimulation (Pirulli et al., 2013), while evidence for the effects of online tDCS stimulation remained mixed (Nitsche et al., 2003). Nevertheless, direct comparison between online and offline tDCS stimulations was encouraged as it enables mapping of boundary conditions of tDCS in the modulation of the rTPJ in facial emotional mimicry.

Although the implementation of the tDCS protocol in this study was largely based on previous well-confirmed research, there are some limitations concerning this method. First, the lack of stimulation of control area and/or implementation of a control task, which is very common in previous studies with a between-subjects design (e.g., Santiesteban et al., 2012; Mai et al., 2016), prevents the current study from being a precise investigation to verify the specificity of the rTPJ. The second limitation is the lack of a blind check. However, based on the fact that when current is delivered even at 1 mA, sham stimulation is reliable for blind participants (Gandiga et al., 2006; Ambrus et al., 2012; Woods et al., 2015). We tend to believe that the set of stimulus conditions in the present study should be efficient. Additionally, a random post-experiment interview during debriefing supported this idea by demonstrating that participants believed they were consistently receiving current stimulation. Nevertheless, we admit that only real blind checks can provide sufficient support for this assumption (Martin et al., 2019a). We thus agree that it would be appropriate for future studies to explicitly assess the validity of the blinding procedure. A third possible limitation concerns the size of the electrodes. Although 35 cm^2^ is a widely used size for tDCS, and especially for an rTPJ-focused tDCS study (e.g., Santiesteban et al., 2012; Mai et al., 2016), caution should be expressed for the possible interfering factors because of the relatively large size. A promising way is to employ larger numbers of smaller electrodes (i.e., 25 mm^2^) to improve the specificity of stimulation, as indicated by a recent review (Solomons and Shanmugasundaram, 2020).

In summary, the present study demonstrates that the rTPJ does not serve as a neural candidate for facial emotional mimicry but acts as the neural substrates for ingroup bias and plays a role in differentiating between ingroup and outgroup members in emotional mimicry. This finding highlights the role of the rTPJ in the modulation of intergroup bias in social cognition.

## Data Availability Statement

The raw data supporting the conclusions of this article will be made available by the authors, without undue reservation.

## Ethics Statement

The studies involving human participants were reviewed and approved by Institutional Review Board of the Department of Psychology, Renmin University of China. The patients/participants provided their written informed consent to participate in this study.

## Author Contributions

SP and PH designed the study. SP, BK, and LZ collected and analyzed the data. SP wrote the draft. SP, BK, and PH revised the draft. All authors contributed to the article and approved the submitted version.

## Conflict of Interest

The authors declare that the research was conducted in the absence of any commercial or financial relationships that could be construed as a potential conflict of interest.
